# Estimation of Surgery Durations Using Machine Learning Methods-A Cross-Country Multi-Site Collaborative Study

**DOI:** 10.3390/healthcare10071191

**Published:** 2022-06-25

**Authors:** Sean Shao Wei Lam, Hamed Zaribafzadeh, Boon Yew Ang, Wendy Webster, Daniel Buckland, Christopher Mantyh, Hiang Khoon Tan

**Affiliations:** 1 Health Services and Systems Research, Duke-NUS Medical School, Health Services Research Centre, Singapore Health Services, Singapore 169856, Singapore; ang.boon.yew@singhealth.com.sg; 2 SingHealth Duke-NUS Global Health Institute, SingHealth Duke-NUS Academic Medical Centre, Singapore 168753, Singapore; tan.hiang.khoon@singhealth.com.sg; 3 Department of Surgery, Duke University School of Medicine, Durham, NC 27710, USA; hz116@duke.edu (H.Z.); wendy.webster@duke.edu (W.W.); dan.buckland@duke.edu (D.B.); christopher.mantyh@duke.edu (C.M.); 4 Department of Biostatistics and Bioinformatics, Duke University School of Medicine, Durham, NC 27710, USA; 5 Thomas Lord Department of Mechanical Engineering and Materials Science, Duke University, Durham, NC 27708, USA; 6 Division of Surgery and Surgical Oncology, Singapore General Hospital, Singapore 168753, Singapore

**Keywords:** surgical durations, machine learning, data sharing, multi-country, multi-site, big data analytics

## Abstract

The scheduling of operating room (OR) slots requires the accurate prediction of surgery duration. We evaluated the performance of existing Moving Average (MA) based estimates with novel machine learning (ML)-based models of surgery durations across two sites in the US and Singapore. We used the Duke Protected Analytics Computing Environment (PACE) to facilitate data-sharing and big data analytics across the US and Singapore. Data from all colorectal surgery patients between 1 January 2012 and 31 December 2017 in Singapore and, 1 January 2015 to 31 December 2019 in the US were used, and 7585 cases and 3597 single and multiple procedure cases from Singapore and US were included. The ML models were based on categorical gradient boosting (CatBoost) models trained on common data fields shared by both institutions. The procedure codes were based on the Table of Surgical Procedure (TOSP) (Singapore) and the Current Procedural Terminology (CPT) codes (US). The two types of codes were mapped by surgical experts. The CPT codes were then transformed into the relative value unit (RVU). The ML models outperformed the baseline MA models. The MA, scheduled durations and procedure codes were found to have higher loadings as compared to surgeon factors. We further demonstrated the use of the Duke PACE in facilitating data-sharing and big data analytics.

## 1. Introduction

Operating Rooms (ORs) account for a significant proportion of a hospital’s total revenue and about 40% of the hospital’s total expenses. [1] With a global estimate of 312.9 million surgeries performed each year, the effective planning of OR resources is an essential process that has a large impact on hospital surgical processes worldwide [2]. The scheduling of surgical procedures plays an important role in the OR planning process and has a direct impact on resource utilization, patient outcomes and staff welfare. Optimal scheduling of OR slots for surgeries prevents under-utilization of costly surgical resources as well as delays which cause unfavorable waiting times. Overtime resulting from sub-optimal schedules also leads to staff dissatisfaction as well as burnout from long working hours [3,4]. One key factor required in optimal scheduling of OR slots is the accurate prediction of surgery duration [3,5]. However, the variability in patients’ conditions and the type of surgical procedures and techniques required and the uncertainties around these patient and provider related factors present challenges for the prediction of surgery durations [6,7]. 

In recent years, many studies around the world have reported the use of various machine-learning (ML) methods to accurately predict surgery duration [8,9,10]. Linear regression techniques have been explored using patient and surgical factors and have reported the importance of such variables in predicting the total surgical procedure time [11]. Distributional modelling methods such as Kernel Density Estimation (KDE) [12] as well as log-normal distributions [13] were also demonstrated to be able to effectively predict surgery duration. More complex methods such as heteroscedastic neural network regression combined with expressive drop-out regularized neural networks have also been shown to have good performance [14]. Ensemble tree-based methods such as random forests were also used to predict surgery duration and cross validation showed that it outperformed other methods, reducing the mean absolute percentage error by 28%, when compared to current hospital estimation approaches [15]. A multi-center study based on two large European teaching hospitals has also demonstrated the use of a parsimonious lognormal modelling approach to improve the estimation of surgery duration and OR efficiency across more than one hospital [13].

The significance of multiple factors affecting surgical duration predictions may vary across hospital systems due to differences in case scheduling practices, surgical techniques and intraoperative processes. A broader understanding of the differences in the characteristics of the scheduling processes as well as scheduling behavior will lead to further insights into improving such estimations. In order to determine hospital level differences for estimating surgery duration, there is a need to go beyond simple distributional approaches to understand the multifactorial effects influencing the length of surgery durations across multiple sites. ML models such as gradient boosted trees, which utilize error residuals to improve the performance of ensemble models, have also been reported for other clinical prediction models, such as anterior chamber depth (ACD) in cataract surgery [16].

Although previous studies have examined the use of various ML prediction modelling methods to provide more accurate estimates, most of these studies offer results that are specific to a single institution with a minority that derived prediction models validated with external institutional data, albeit from the same country [3,6,10,11,13,14,17]. Similar to other use cases where prediction models are developed based on an extensive use of real-world data, a key reason resulting in the difficulty of conducting multi-site across multiple countries is the lack of data sharing and of governance infrastructure to support collaborative work. This impediment can be further magnified when the sharing of data has to occur over multiple jurisdictions. This has resulted in a scarcity of published studies that can cover multi-institutional data across countries or continents, thereby reducing the external validity and generalizability of the prediction models.

In this study, we aim to determine the performance of current surgery case duration estimations and the use of machine learning models to predict surgery duration across two large teaching hospitals in the United States and Singapore. To facilitate deep collaboration between both hospitals and the sharing of large-scale datasets required for the development of the ML models, we will introduce the use of Duke Protected Analytics Computing Environment (PACE) [18]. PACE is a collaborative platform for facilitating data-sharing and analysis across both healthcare institutions. This study has demonstrated value in the use of PACE for a cross border and multi-institutional studies in the evaluation of surgical durations across institutions.

## 2. Materials and Methods

The two study hospitals SH-1 and SH-2 described in this study are from Singapore, a city-state in Southeast Asia, and Durham, North Carolina, a state in the United States, respectively. SH-1 is the Singapore General Hospital (SGH), which is one of the largest comprehensive public hospitals in Singapore under the Singapore Health Services (SingHealth) public healthcare cluster. SGH is a tertiary multidisciplinary academic hospital which comprises more than 30 clinical disciplines and approximately 1700 inpatient beds and provides acute and specialist care to over one million patients per year [19]. The hospital saw more than 25,000 surgeries in 2019. SH-2 is Duke University Hospital, a full-service tertiary and quaternary care hospital that is part of the Duke University Health System in Durham, North Carolina. Duke University Hospital has 957 inpatient beds, 51 operating rooms, an endo-surgery center and an ambulatory surgery center with nine operating rooms. The hospital offers multidisciplinary care and serves as a regional emergency/trauma center where 42,554 patients were admitted in 2020 [20,21].

Ethics approval for the study was exempted by both the SingHealth’s Centralized Institutional Review Board (SingHealth CIRB Reference: 2018-2558) and Duke Institutional Review Board (Duke IRB Reference: Pro00104275) for both study hospitals.

### 2.1. Cross Country Collaborative Platform

The Duke PACE [22] is a secured virtualized network environment where researchers can collaborate and perform analysis with protected health information. PACE simplifies the process of obtaining and sharing protected data from the electronic medical record (EMR) systems. Datasets from both study hospitals are shared and analyzed jointly by the study team through the PACE system. The use of PACE requires video-based training and a rigorous account request and approval process for Duke University employees and affiliates. Data loaded in PACE has to be HIPAA compliant. Ethics approval or exemption has to be given by the ethics review boards of the respective study hospitals.

Data was extracted from the SH-1 EMR system based on the Sunrise Clinical Manager, Allscripts [23], extracted through the enterprise data warehouse, electronic Health Intelligence System-eHIntS [24]. Data from the SH-2 EMR system were extracted from the Duke Health enterprise data warehouse and Duke’s Maestro Care (Epic) EMR system [25]. These data were loaded into PACE and then served through a secured Duo multifactor authentication gateway [26] for access by collaborators across the two countries with approved network IDs. The analysis was performed with Python 3.6, Python Software Foundation [27], with the required packages loaded into the PACE environment. The hardware provisioned in PACE for this study was Intel(R) Xeon(R) Gold 6252 CPU @ 2.10GHz (2 processors) (Intel Corporation) and 32 GB of RAM running on Windows 10 Enterprise operating system (Microsoft Corporation). Access was time-bound based on the approved period of study according to the respective ethics review boards’ decisions.

### 2.2. Descriptive Analysis

We performed a retrospective analysis of all patients who had undergone colorectal surgery between 1 January 2012 and 31 December 2017 for SH-1 and 1 January 2015 to 31 December 2019 for SH-2. Common data fields were mapped between datasets from both study sites and used in the study. Patient demographics included age, gender, height, weight and body mass Index (BMI). Surgery related factors included surgery procedure codes, number of procedures done in the surgery, first and second surgeons codes, principal anesthetist codes, anesthesia type (local, general or regional anesthesia), patient case type (inpatient or day surgery), OR location, OR code and ASA scores. The Table of Surgical Procedures (TOSP) is a categorical variable used for billing purposes [28]. TOSP codes provide some information on the complexity of the procedure codes used in SH-1, where higher levels represent greater complexity. SH-2 uses the categorical Current Procedural Terminology (CPT) codes that similarly show the complexity of the procedures and services [29]. The TOSP and CPT codes for colorectal surgeries used in this study were mapped by the surgical domain experts and shown in Table A1, Appendix A. The list of mapped fields across the two institutions is shown in Table 1. In SH-2, the categorical CPT codes per case were transformed into the relative value unit (RVU) which is a single continuous variable (see Table 2). The RVU is a consensus driven billing indicator that can serve as a proxy for procedure workload and replace CPT codes as a more informative feature of surgical duration predictions [30,31]. The scheduled/listing duration for the surgical case as well as the moving average (MA) durations were also included.

**Figure 1:**
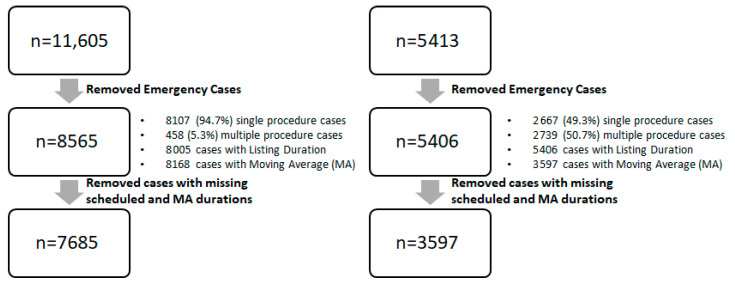
Data-cleaning process (MA: Moving Average).

A total of 7585 cases and 3597 cases from SH-1 and SH-2 were included respectively. The data cleaning process is shown in Figure 1 for both SH-1 and SH-2. The mean duration for SH-1 and SH-2 were 102 min and 128 min, respectively. The mean patient age across both sites were 54.8 and 54.4 years, respectively.

### 2.3. Moving Average Estimation

The existing EMR systems for both SH-1 and SH-2 surgical case management both adopt a moving average (MA) prediction of historical surgery durations to provide an estimated surgery duration for each surgery case. SH-1 uses Allscripts [23], whilst SH-2 uses the EPIC system [25]. The MA algorithm calculates a historical moving average of actual surgery duration by grouping surgical procedure codes and surgeon codes over a specified period of time. The OR schedulers, who schedule surgery cases into the respective EMR systems, are able to override the estimates with their own estimated durations.

The historical moving average of the actual case length for each procedure and surgeon code combination was used as the prediction for the next surgery of the same procedure code and conducted by the same surgeon. If there are less than five cases for a particular surgeon and procedure code combination, the MA of the surgical duration for that particular procedure (regardless of surgeon) is utilized. If the data is insufficient for this grouping, no MA estimate will be provided and the scheduler will have to provide an estimate instead. If there are sufficient data for the MA estimates, data below the 10th percentile and above the 90th percentile will be excluded from the MA calculation. If there are no manual overrides on the MA prediction, the MA-based duration is then recorded as the scheduled duration and is used to schedule cases in the system. The existing estimates as well as the scheduled durations will be used as the baseline against which new predictions developed by machine learning algorithms in this study will be compared.

Cases which were listed as emergency cases as well as those with missing actual surgery duration were excluded for the study. Both single and multiple procedure surgeries were included. The data cleaning process is summarized in Figure 1. Cases with missing values for either scheduled duration or MA duration were excluded. Outcome metrics were compared by available cases by individual duration type, as well as a common set of valid cases.

The outcome of interest was the difference between the predicted and the actual surgery durations for each surgical case. Surgery duration was defined as the time taken between the point when the patient is wheeled into the OR and when the patient is wheeled out of the OR. For each comparison, we compared the Root Mean Squared Error (RMSE), Mean Absolute Error (MAE), Mean Absolute Percentage Error (MAPE) and the percentage of cases within 20% of scheduled duration with the predicted duration.

The predictive models were generated with the categorical gradient boosting (CatBoost, version 0.2.1) [32] package in Python 3.6 [27]. The dataset of each study hospital was split into 80% for training and 20% for testing. The models were trained on the common data fields (Patient and Surgery factors) shared by both datasets, with different permutations of additional key variables such as the Moving Average, Scheduled/Listing Duration and TOSP Code/RVU. The CatBoost models with features listed in Table 2 were compared. SH-1’s models were trained and tested on SH-1’s dataset and SH-2’s models were trained and tested on SH-2’s dataset.

Hyperparameter optimization was conducted using a grid search on a four-fold cross validation performed to determine the optimal parameters for the models. The following parameters were used for the CatBoost models-Number of Iterations: 200; Maximum Tree Depth: 5, 6, 7, 8, 9, 10, 11, Learning Rate: 0.01, 0.03, 0.05, 0.1, 0.2, 0.3; Loss Function: Root Mean Square Error (RMSE). The parameters which provided the lowest cross validation RMSE score were chosen for the final model. Feature importance for the CatBoost models were evaluated based on the amount that the prediction value changes with respect to a change in the predictor variable [32].

## 3. Results

### Scheduler and System Average Performance

**Figure 2:**
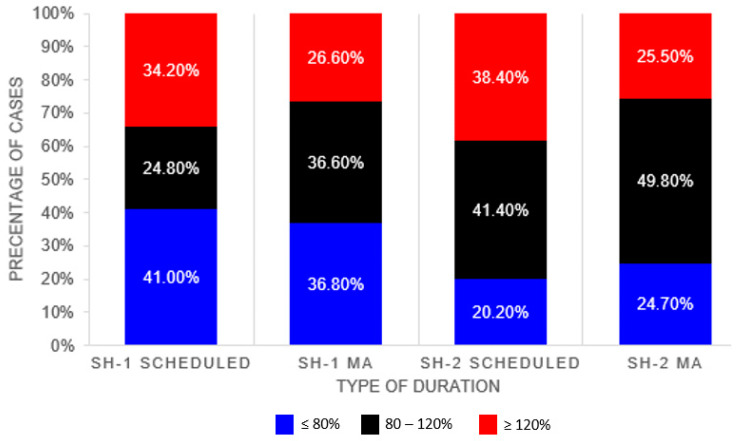
Proportion of Cases within 20% of Actual Durations.

Table 3 compares the performance of the scheduled duration and the MA duration against the actual surgery duration. The MA algorithm provides better performance across all evaluation metrics for both datasets as compared to the scheduled durations. Proportion of cases with the actual duration falling within 80–120% of the listed duration is higher in SH-2. Higher proportion of cases were found to be overestimated in the SH-2 dataset, whereas for SH-1 (>80% of actual duration) higher proportion of cases were found to be underestimated (<80% of actual duration) (see Figure 2).

Table 4 and Table 5 show the performance of the various SH-1 and SH-2 predictive models based on the test dataset. The results showed that with the ML-based models (Models 0–5), SH-1 could at least predict 40% of cases accurately within +/−20% of the actual duration, while SH-2 could at least predict approximately 50% of its cases within +/−20% of the actual duration. Based on the +/−20% prediction band, the ML-based models in both hospitals showed better prediction accuracy than the existing MA models that each individual hospital uses.

In SH-1, Model 5 showed the best performance as shown in Table 4. Model 4 has slightly higher MAE, MAPE and RMSE, as compared to Model 5, but it shows a better prediction accuracy (within +/−20% deviation from the actual duration). Nonetheless, both Models 4 and 5 have at least a 5% higher prediction accuracy than that of the MA. Among the five models in SH-2, Table 5 shows that Model 5 has the best performance, with 56.11% of its predictions falling within +/−20% of the actual duration. Model 5 prediction accuracy (within +/−20%) is 7.78% higher than that of the MA. Model 5 also has the lowest RMSE, MAE and MAPE at 38.48%, 23.61% and 23.36%, respectively.

**Figure 3:**
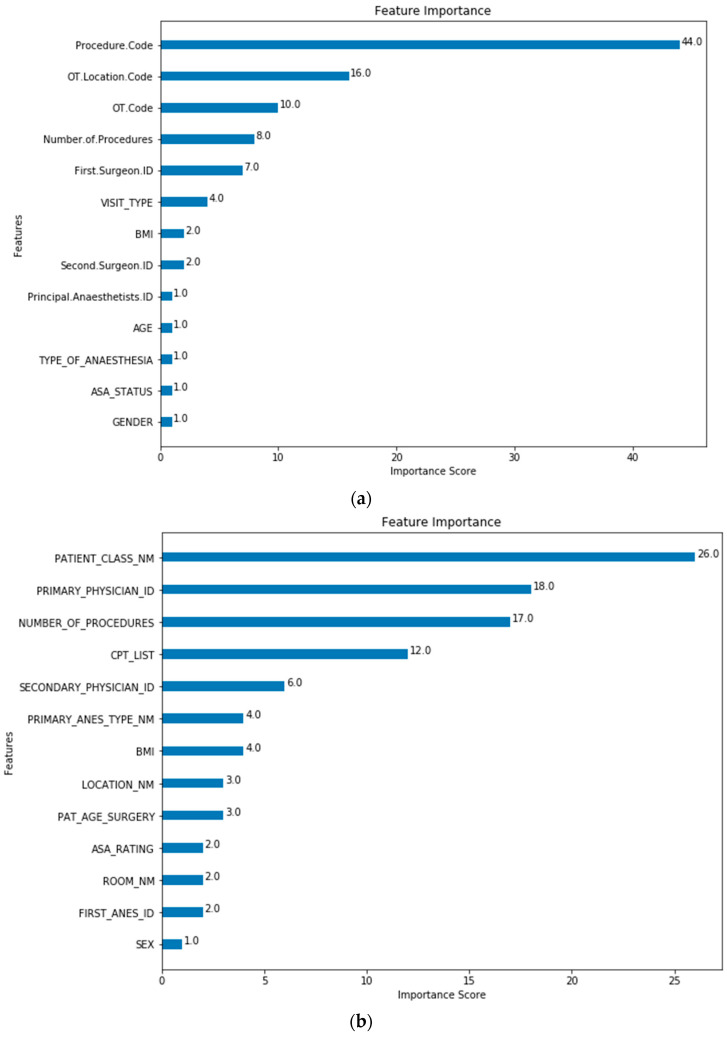
Model 0 Feature Importance for (**a**) SH-1; (**b**) SH-2. (Note: Refer to Table 1 for feature mapping between SH-1 and SH-2).

**Figure 4:**
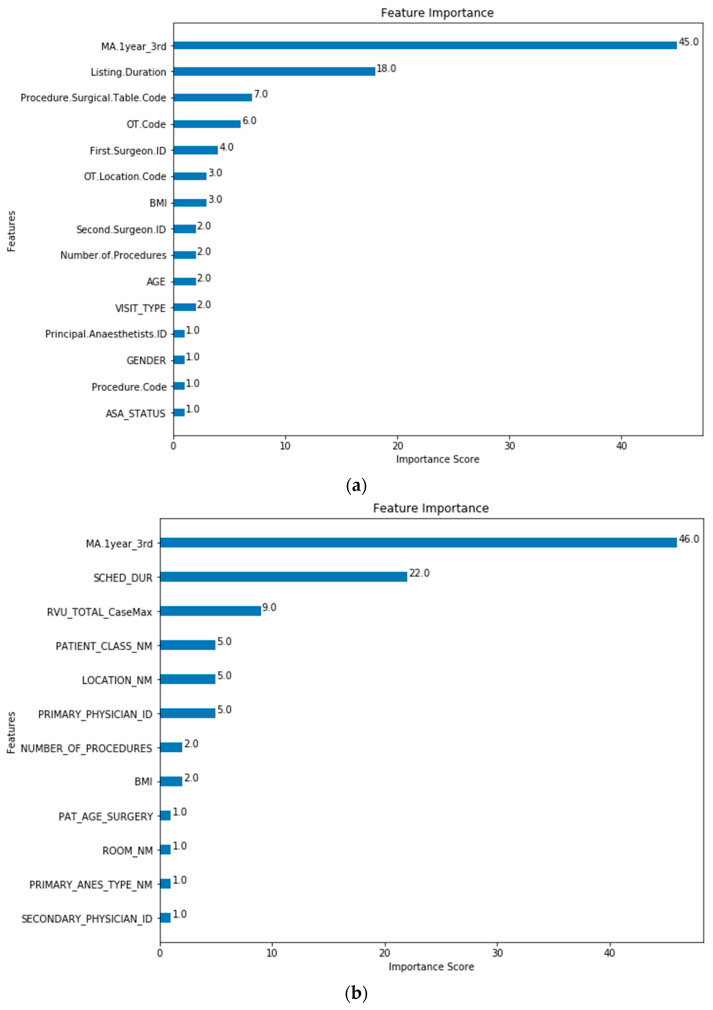
Model 5 Feature Importance for (**a**) SH-1 (Procedure.Surgical.Table.Code is “Procedure Code”; (**b**) SH-2 (RVU_TOTAL_CaseMax is “CPT List”). (Note: Refer to Table 1 for feature mapping between SH-1 and SH-2).

The feature importance of each predictor variable is averaged across all of the decision trees within the model. The best performing models (Model 5 for both SH-1 and SH-2) were used to plot the feature importance shown in Figure 3 and Figure 4.

## 4. Discussion

The objective of this study was to explore and determine the performance of current surgery case duration estimations and the use of machine learning models to predict surgery duration across two large tertiary healthcare institutions located in the United States and Singapore. The two healthcare institutions have different EMR systems, coding and representation of surgical details such as surgical procedure codes. The Table of Surgical Procedure (TOSP) codes [28] were used in SH-1 whilst the Current Procedural Terminology (CPT) [29] codes were used in SH-2. The two types of codes were mapped by surgical domain experts. The mapping table is given in Appendix A.

The validation results showed that, in both study sites, the simple MA-based predictions outperform the scheduled duration provided by the OR schedulers across RMSE, MAE, MAPE and proportion of cases within 80–120% of the scheduled actual duration. Every minute of improved duration estimates would help in improving the efficiency of OR performance [15,33]. MA-based predictions have been frequently reported in the literature. Similar to the existing literature [3,6,34], both hospitals have been using simple MA-based methods, such as Last-5 [6], which uses the average of the most recent five cases in the relevant history for the prediction. Simple MA methods can be accurate in the estimation of surgery durations across multiple sites.

The baseline machine-learning (ML) models which considered patient, surgeon and surgery related factors without the MA (Model 0) show improved performance for SH-1 across all metrics against the scheduled durations and MA estimates. Extending from the baseline model, Models 2, 4 and 5 included the MA features to improve the performance and generalizability for the predictions. The improved performance of ML-based models is similar to results that were recently reported [8,10,17,35]. For both SH-1 and SH-2, the majority of the contributions to the model was based on the MA and scheduled duration. For SH-1, the next five variables with the highest contributions are: Procedure Surgical Table Code, OT Code, First Surgeon ID, OT Location Code and BMI. At SH-2, the most significant factors are also MA and scheduled duration. whilst the next five variables with the highest contributions are RVU, Patient Class, Primary Physician ID and OR Location and Number of Procedures. In both SH-1 and SH-2, MA, scheduled duration and, TOSP Code (for SH-1) or RVU (for SH-2), have higher loading in the model as compared to surgeon factors. However, the order of importance of the other variables differs slightly between the two sites. For both sites, the variables describing the complexity of the surgery (TOSP Code in SH-1 and RVU in SH-2) have relatively higher loadings in the prediction models. The presence of the MA, Scheduled/Listing and Procedure Surgical Table Code and RVU only for Model 5 may have resulted in the better performance of this model. All these features have the highest contributions in Model 5 feature importance for SH-1 and SH-2 as shown in Figure 4.

As the study sites utilized similar datasets across different study periods, there may be concerns about model bias. However, for both sites during the study horizon, there were no significant shifts in the surgical procedures for colorectal procedures and the design of the EMR systems and the extract-transform-load (ETL) system within the enterprise data warehouse across both sites. Moreover, each hospital has its own trained CatBoost ML model [32] so different periods in one model will not affect the other. The framework using CatBoost ML models has been tuned to provide the best prediction models based on the lowest cross validation RMSE. This result can be further evaluated in future studies in collaboration with more study sites. The collaborative PACE platform [22] has been shown to facilitate such study across two different jurisdictions.

Electronic health record (EHR) data are extremely sensitive and valuable and require a protected environment to work in. This can be difficult and time consuming to achieve even in one institute. Duke PACE [22] provides a secured and protected environment to query and store these data and perform advanced analysis. This study demonstrates that PACE can provide the platform for this study to share EHR data between the two institutes of the two countries and facilitates the use of advanced machine-learning tools to predict surgical durations. Similar features were used in the prediction models developed at both sites (see Table 1). This study shows a viable alternative to facilitate future collaboration between institutes around the world. The collaboration through PACE demonstrated the feasibility in data sharing, validating the hypothesis and collaborative development of analytical models in order to support better clinical decision that can improve system, process and patient outcomes.

## 5. Conclusions

In this study, we compared the performance of existing MA-based estimates with novel ML-based predictive models for surgery durations across two large tertiary healthcare institutions. The ML-based models which considered additional patient, surgeon and surgery related factors show improved performance over both the MA-based method and the scheduled durations across multiple accuracy metrics. The ML-based models can be deployed in place of the existing MA-based estimates. Additional patient-related factors (e.g., comorbidities) could potentially help to further improve the accuracies of the predictions.

We further demonstrated the use of the Duke PACE as the collaborative platform for facilitating data-sharing and analysis across both healthcare institutions for cross border and cross-institutional studies. Duke PACE was able to overcome the impediments in data sharing and governance policies to support collaborative work across multiple jurisdictions.

## Figures and Tables

**Table 1:** Field mapping between SH-1 and SH-2.

SN	SH-1 Data Fields	SH-2 Data Fields
1	OT Code	Room
2	Actual Duration	In-Out Duration
3	First Surgeon Department Code	Service Type
4	Priority of Operation	Case Class
5	Department Code	Division
6	OT Location Code	Location
7	Procedure Code	CPT List
8	Type of Anesthesia	Primary Anesthesia Type
9	ASA Status	ASA Rating
10	Age	Patient Age
11	Gender	Sex
12	Visit Type	Patient Class
13	BMI	BMI
14	Height	Height
15	Weight	Weight
16	First Surgeon ID	Primary Physician ID
17	Second Surgeon ID	Secondary Physician ID
18	Principal Anesthetist ID	First Anesthetist ID
19	MA 1 year_3rd	MA 1 year_3rd (calculated)
20	Number of Procedures	Number of Procedures
21	Number of Panels	Number of Panels
22	Multiple Procedure Codes	Sorted CPT List
23	Listing Duration	Scheduled Duration

Notations: OT: Operating Theatre; CPT: Current Procedure Terminology (transformed into Relative Value Units); ASA: American Society of Anesthesiology; BMI: Body Mass Index; ID: Unique identifier; MA: Moving Average.

**Table 2:** List of CatBoost models compared (List of features present in each model are shown in Table A2 in the Appendix A).

Name	Features Considered
Model 0	Baseline Model which considered patient and surgery factors only
Model 1	Baseline Model + RVU/Procedure Surgical Table Code
Model 2	Baseline Model + Moving Average
Model 3	Baseline Model + Scheduled Duration
Model 4	Baseline Model + Moving Average + Scheduled Duration
Model 5	Baseline Model + Moving Average + Scheduled Duration + RVU/Procedure Surgical Table Code

**Table 3:** Performance of scheduled vs. MA duration.

	SH-1		SH-2	
	Scheduled	MA	Scheduled	MA
**N (cases)**	7685	7685	3597	3597
**RMSE**	61.5	51.5	57.5	48.2
**MAE (mins)**	37.7	29.2	34.8	29.5
**MAPE (%)**	7.49%	2.40%	15.91%	5.54%
**<=80%**	41.0%	36.8%	20.2%	24.7%
**80–120%**	24.8%	36.6%	41.4%	49.8%
**>=120%**	34.2%	26.6%	38.4%	25.5%

RMSE: Root Mean Squared Error; MAE: Mean Absolute Error; MAPE: Mean Absolute Percentage Error.

**Table 4:** SH−1 Accuracy & Error Metrics Comparison of Models.

Model	Percentage within +\−20%	RMSE	MAE	MAPE
**Listing**	24.68%	62.31	37.505	65.57%
**MA**	37.66%	55.16	28.844	46.85%
**Model 0**	40.31%	48.15	26.323	36.74%
**Model 1**	43.15%	47.88	25.221	34.61%
**Model 2**	43.28%	47.30	24.938	35.56%
**Model 3**	41.34%	46.26	25.426	34.97%
**Model 4**	42.89%	45.30	24.325	34.50%
**Model 5**	44.06%	45.18	23.986	34.40%

RMSE: Root Mean Squared Error; MAE: Mean Absolute Error; MAPE: Mean Absolute Percentage Error.

**Table 5:** SH-2 Accuracy & Error Metrics Comparison of Models.

Model	Percentage within +\−20%	RMSE	MAE	MAPE
**Listing**	43.06%	53.57	32.167	27.63%
**MA**	48.33%	45.39	28.19	27.30%
**Model 0**	49.86%	50.845	30.492	27.23%
**Model 1**	52.78%	38.817	24.412	23.83%
**Model 2**	55.42%	40.9	25.529	24.90%
**Model 3**	55.28%	43.208	26.18	24.54%
**Model 4**	55.42%	39.367	24.518	23.79%
**Model 5**	56.11%	38.482	23.61	23.36%

RMSE: Root Mean Squared Error; MAE: Mean Absolute Error; MAPE: Mean Absolute Percentage Error.

## Data Availability

The data presented in this study may be available on request from the corresponding author subject to legal or collaboration agreements. The data are not publicly available due to the proprietary nature of the data.

## Appendix A

**Table A1:** Mapping of CPT to TOSP codes.

Anatomical System	Type of Procedure	CPT Code	TOSP Codes	TOSP Table
Digestive	Hepatectomy	47120	SF815L	4C
Digestive	Hepatectomy	47120	SF813L	5C
Digestive	Hepatectomy	47122	SF809L	7C
Digestive	Hepatectomy	47125	SF812L	6B
Digestive	Hepatectomy	47130	SF812L	6B
Digestive	Appendectomy	44950	SF849A	3B
Digestive	Appendectomy	44950	SF723A	4A
Digestive	Appendectomy	44960	SF849A	3B
Digestive	Appendectomy	44960	SF723A	4A
Digestive	Appendectomy	44970	SF849A	3B
Digestive	Appendectomy	44970	SF723A	4A
Digestive	Colorectal	44140	SF701C	6C
Digestive	Colorectal	44140	SF803C	5C
Digestive	Colorectal	44140	SF806C	5C
Digestive	Colorectal	44143	SF808R	5C
Digestive	Colorectal	44144	SF808R	5C
Digestive	Colorectal	44145	SF805R	6C
Digestive	Colorectal	44145	SF703R	6C
Digestive	Colorectal	44145	SF807R	6B
Digestive	Colorectal	44146	SF805R	6C
Digestive	Colorectal	44146	SF703R	6C
Digestive	Colorectal	44146	SF807R	6B
Digestive	Colorectal	44147	SF703R	6C
Digestive	Colorectal	44150	SF804C	6A
Digestive	Colorectal	44150	SF712C	6A
Digestive	Colorectal	44151	SF804C	6A
Digestive	Colorectal	44151	SF712C	6A
Digestive	Colorectal	44160	SF803C	5C
Digestive	Colorectal	44204	SF701C	6C
Digestive	Colorectal	44204	SF803C	5C
Digestive	Colorectal	44204	SF806C	5C
Digestive	Colorectal	44205	SF803C	5C
Digestive	Colorectal	44206	SF808R	5C
Digestive	Colorectal	44207	SF805R	6C
Digestive	Colorectal	44207	SF703R	6C
Digestive	Colorectal	44207	SF807R	6B
Digestive	Colorectal	44208	SF805R	6C
Digestive	Colorectal	44208	SF703R	6C
Digestive	Colorectal	44208	SF807R	6B
Digestive	Colorectal	44210	SF712C	6A
Digestive	Colorectal	44210	SF804C	6A
Digestive	Esophagectomy	43101	SF802E	5B
Digestive	Esophagectomy	43107	SF809E	7B
Digestive	Esophagectomy	43108	SM702L	7C
Digestive	Esophagectomy	43112	SF809E	7B
Digestive	Esophagectomy	43112	SM702L	7C
Digestive	Esophagectomy	43113	SF809E	7B
Digestive	Esophagectomy	43113	SM702L	7C
Digestive	Esophagectomy	43116	SF806E	7C
Digestive	Esophagectomy	43117	SF804E	6B
Digestive	Esophagectomy	43117	SF809E	7B
Digestive	Esophagectomy	43118	SF804E	6B
Digestive	Esophagectomy	43118	SF809E	7B
Digestive	Esophagectomy	43121	SF804E	6B
Digestive	Esophagectomy	43122	SF804E	6B
Digestive	Esophagectomy	43123	SF804E	6B
Digestive	Esophagectomy	43124	SF812E	3A
Digestive	Esophagectomy	43124	SF806E	7C
Digestive	Pancreatectomy	48120	SF705P	4C
Digestive	Pancreatectomy	48120	SF706P	5A
Digestive	Pancreatectomy	48140	SF708P	5B
Digestive	Pancreatectomy	48145	SF809P	7C
Digestive	Pancreatectomy	48145	SF712P	5C
Digestive	Pancreatectomy	48146	SF703P	7A
Digestive	Pancreatectomy	48146	SF704P	7A
Digestive	Pancreatectomy	48148	SF807B	5C
Digestive	Pancreatectomy	48150	SF809P	7C
Digestive	Pancreatectomy	48152	SF809P	7C
Digestive	Pancreatectomy	48153	SF809P	7C
Digestive	Pancreatectomy	48154	SF809P	7C
Digestive	Pancreatectomy	48155	SF809P	7C
Digestive	Colorectal	44155	SF712C	6A
Digestive	Colorectal	44155	SF804C	6A
Digestive	Colorectal	44155	SF805C	6B
Digestive	Colorectal	44156	SF805C	6B
Digestive	Colorectal	44157	SF805C	6B
Digestive	Colorectal	44157	SF713C	6C
Digestive	Colorectal	44158	SF713C	6C
Digestive	Colorectal	44211	SF713C	6C
Digestive	Colorectal	44212	SF712C	6A
Digestive	Colorectal	44212	SF804C	6A
Digestive	Colorectal	44212	SF805C	6B
Digestive	Colorectal	45110	SF845A	6B
Digestive	Colorectal	45110	SF805R	6C
Digestive	Colorectal	45111	SF805C	6B
Digestive	Colorectal	45111	SF701R	5C
Digestive	Colorectal	45112	SF807R	6B
Digestive	Colorectal	45113	SF807R	6B
Digestive	Colorectal	45114	SF701R	5C
Digestive	Colorectal	45116	SF701R	5C
Digestive	Colorectal	45119	SF807R	6B
Digestive	Colorectal	45120	SF803R	5C
Digestive	Colorectal	45120	SF700R	5C
Digestive	Colorectal	45121	SF803R	5C
Digestive	Colorectal	45126	SF703R	6C
Digestive	Colorectal	45126	SF808R	5C
Digestive	Colorectal	45126	SF805A	6B
Digestive	Colorectal	45130	SF700R	5C
Digestive	Colorectal	45135	SF700R	5C
Digestive	Colorectal	45160	SF701R	5C
Digestive	Colorectal	45395	SF805C	6B
Digestive	Colorectal	45395	SF805C	6B
Digestive	Colorectal	45397	SF713C	6C
Digestive	Colorectal	45402	SF701R	5C
Digestive	Colorectal	45550	SF701R	5C
Endocrine	Thyroid	60200	SJ801T	3B
Endocrine	Thyroid	60210	SJ802T	4A
Endocrine	Thyroid	60212	SJ802T	4A
Endocrine	Thyroid	60220	SJ804T	6A
Endocrine	Thyroid	60220	SJ802T	4A
Endocrine	Thyroid	60225	SJ804T	6A
Endocrine	Thyroid	60225	SJ802T	4A
Endocrine	Thyroid	60240	SJ803T	5C
Endocrine	Thyroid	60240	SJ703T	6C
Endocrine	Thyroid	60252	SJ702T	6A
Endocrine	Thyroid	60254	SJ702T	6A
Endocrine	Thyroid	60260	SJ702T	6A
Endocrine	Thyroid	60270	SJ702T	6A
Endocrine	Thyroid	60271	SJ702T	6A
Reproductive	Hysterectomy/Myomectomy	58140	SI816U	3B
Reproductive	Hysterectomy/Myomectomy	58146	SI815U	5A
Reproductive	Hysterectomy/Myomectomy	58150	SI803U	4A
Reproductive	Hysterectomy/Myomectomy	58150	SI804U	5C
Reproductive	Hysterectomy/Myomectomy	58150	SI805U	5C
Reproductive	Hysterectomy/Myomectomy	58150	SI812U	5C
Reproductive	Hysterectomy/Myomectomy	58152	SI702U	4C
Reproductive	Hysterectomy/Myomectomy	58180	SI802U	4A
Reproductive	Hysterectomy/Myomectomy	58210	SI825U	5C
Reproductive	Hysterectomy/Myomectomy	58210	SI827U	5A
Reproductive	Hysterectomy/Myomectomy	58210	SI828U	4A
Reproductive	Hysterectomy/Myomectomy	58240	SI824U	6B
Reproductive	Hysterectomy/Myomectomy	58260	SI837U	4A
Reproductive	Hysterectomy/Myomectomy	58260	SI713V	4A
Reproductive	Hysterectomy/Myomectomy	58262	SI723U	4B
Reproductive	Hysterectomy/Myomectomy	58263	SI721U	4B
Reproductive	Hysterectomy/Myomectomy	58270	SI713V	4A
Reproductive	Hysterectomy/Myomectomy	58290	SI837U	4A
Reproductive	Hysterectomy/Myomectomy	58290	SI713V	4A
Reproductive	Hysterectomy/Myomectomy	58291	SI723U	4B
Reproductive	Hysterectomy/Myomectomy	58292	SI721U	4B
Reproductive	Hysterectomy/Myomectomy	58294	SI713V	4A
Reproductive	Hysterectomy/Myomectomy	58541	SI713U	4B
Reproductive	Hysterectomy/Myomectomy	58542	SI713U	4B
Reproductive	Hysterectomy/Myomectomy	58543	SI712U	5A
Reproductive	Hysterectomy/Myomectomy	58544	SI712U	5A
Reproductive	Hysterectomy/Myomectomy	58545	SI709U	3C
Reproductive	Hysterectomy/Myomectomy	58546	SI700O	4B
Reproductive	Hysterectomy/Myomectomy	58548	SI800O	5C
Reproductive	Hysterectomy/Myomectomy	58548	SI804O	4A
Reproductive	Hysterectomy/Myomectomy	58550	SI718U	4B
Reproductive	Hysterectomy/Myomectomy	58552	SI718U	4B
Reproductive	Hysterectomy/Myomectomy	58553	SI718U	4B
Reproductive	Hysterectomy/Myomectomy	58554	SI718U	4B
Reproductive	Hysterectomy/Myomectomy	58570	SI713U	4B
Reproductive	Hysterectomy/Myomectomy	58572	SI712U	5A
Reproductive	Hysterectomy/Myomectomy	58940	SI805O	3B
Reproductive	Hysterectomy/Myomectomy	58951	SI800O	5C
Reproductive	Hysterectomy/Myomectomy	58951	SI711U	6A
Reproductive	Hysterectomy/Myomectomy	58953	SI804O	4A
Reproductive	Hysterectomy/Myomectomy	58954	SI800O	5C
Reproductive	Hysterectomy/Myomectomy	58954	SI804O	4A
Musculoskeletal	THA	27125	SB838H	5C
Musculoskeletal	THA	27130	SB839H	6A
Musculoskeletal	THA	27130	SB723H	6B
Musculoskeletal	THA	27132	SB724H	6C
Musculoskeletal	THA	27134	SB724H	6C
Musculoskeletal	THA	27137	SB724H	6C
Musculoskeletal	THA	27138	SB724H	6C
Kidney	Nephrectomy	50220	SG816K	4B
Kidney	Nephrectomy	50225	SG816K	4B
Kidney	Nephrectomy	50230	SG804K	5C
Kidney	Nephrectomy	50234	SG800K	5C
Kidney	Nephrectomy	50236	SG800K	5C
Kidney	Nephrectomy	50240	SG721K	5C
Kidney	Nephrectomy	50543	SG720K	6A
Kidney	Nephrectomy	50545	SG710K	6A
Kidney	Nephrectomy	50546	SG700K	6A
Kidney	Nephrectomy	50546	SG722K	4C
Kidney	Nephrectomy	50548	SG700K	6A

**Table A2:** List of features present in each CatBoost model.

			Model Number					
SN	SH-1 Data Fields	SH-2 Data Fields	0 (Baseline)	1	2	3	4	5
1	OT Code	Room	✓	✓	✓	✓	✓	✓
2	Actual Duration	In-Out Duration	✓	✓	✓	✓	✓	✓
3	First Surgeon Department Code	Service Type	✓	✓	✓	✓	✓	✓
4	Priority of Operation	Case Class	✓	✓	✓	✓	✓	✓
5	Department Code	Division	✓	✓	✓	✓	✓	✓
6	OT Location Code	Location	✓	✓	✓	✓	✓	✓
7	Procedure Code	CPT List		**✓**				**✓**
8	Type of Anesthesia	Primary Anesthesia Type	✓	✓	✓	✓	✓	✓
9	ASA Status	ASA Rating	✓	✓	✓	✓	✓	✓
10	Age	Patient Age	✓	✓	✓	✓	✓	✓
11	Gender	Sex	✓	✓	✓	✓	✓	✓
12	Visit Type	Patient Class	✓	✓	✓	✓	✓	✓
13	BMI	BMI	✓	✓	✓	✓	✓	✓
14	Height	Height	✓	✓	✓	✓	✓	✓
15	Weight	Weight	✓	✓	✓	✓	✓	✓
16	First Surgeon ID	Primary Physician ID	✓	✓	✓	✓	✓	✓
17	Second Surgeon ID	Secondary Physician ID	✓	✓	✓	✓	✓	✓
18	Principal Anesthetist ID	First Anesthetist ID	✓	✓	✓	✓	✓	✓
19	MA 1year_3rd	MA 1year_3rd (calculated)			**✓**		**✓**	**✓**
20	Number of Procedures	Number of Procedures	✓	✓	✓	✓	✓	✓
21	Number of Panels	Number of Panels	✓	✓	✓	✓	✓	✓
22	Multiple Procedure Codes	Sorted CPT List	✓	✓	✓	✓	✓	✓
23	Listing Duration	Scheduled Duration				**✓**	**✓**	**✓**

Notations: OT: Operating Theatre; CPT: Current Procedure Terminology (transformed into Relative Value Units); ASA: American Society of Anesthesiology; BMI: Body Mass Index; ID: Unique identifier; MA: Moving Average.
